# Liver–muscle metabolic crosstalk: xanthosine as a key effector of broiler myogenesis

**DOI:** 10.1186/s40104-025-01346-y

**Published:** 2026-02-08

**Authors:** Yiwei Chen, Cong Ding, Meijuan Ren, Zhixuan Li, Shiqi Liu, Haoming Sun, Sijia Yu, Qiang Niu, Xingyu Li, Bing Li, Li Li, Xiaojun Yang, Qingzhu Sun

**Affiliations:** College of Animal Science and Technology, Northwest A&F University, Xinong Road No.22, Yangling, Shaanxi 712100 China

**Keywords:** Broilers, Caffeine metabolism, *CYP1A2*, Myoblast, Xanthosine

## Abstract

**Background:**

Nutritional strategies aimed at augmenting growth performance remain a central focus in poultry science. The liver, as a pivotal metabolic organ, exerts profound influence on skeletal muscle development. Nevertheless, the mechanistic interplay between hepatic metabolism and myogenesis has not been fully delineated. Here, by integrating multi-omics analyses with functional validation, we identified xanthosine, a metabolic derivative of hepatic caffeine catabolism, as a previously unrecognized regulator of broiler muscle growth. We further elucidated its mechanistic role in promoting myoblast proliferation.

**Results:**

Comparative phenotypic assessment of high- and low-body-weight broilers revealed substantial differences in breast muscle mass. Metagenomic profiling of cecal microbiota demonstrated only a limited association between microbial composition and body weight. In contrast, untargeted plasma metabolomics uncovered a systemic upregulation of amino acid metabolism in high-body-weight broilers, concomitant with a pronounced activation of caffeine metabolism. Consistently, hepatic transcriptomic profiling revealed marked induction of cytochrome P450 family 1 subfamily A member 2 (*CYP1A2*), encoding a key enzyme catalyzing caffeine catabolism. Integrated KEGG pathway enrichment across metabolomic and transcriptomic datasets highlighted caffeine metabolism as a significantly perturbed pathway. Among its downstream metabolites, plasma xanthosine was robustly elevated in high-body-weight broilers. Functional validation via in ovo injection demonstrated that xanthosine administration significantly augmented post-hatch growth performance by increasing skeletal muscle mass. Mechanistic investigations further established that xanthosine drives myoblast proliferation through activation of the ERK/GSK3β/β-catenin signaling cascade.

**Conclusions:**

Together, these findings delineate a liver–muscle metabolic axis in which hepatic *CYP1A2*-driven caffeine metabolism elevates circulating xanthosine, which in turn acts as a pivotal molecular effector of myogenic growth. This study uncovers a previously unappreciated metabolic mechanism by which hepatic activity orchestrates skeletal muscle development. It also highlights targeted modulation of xanthosine metabolism as a promising strategy to enhance broiler growth performance and production efficiency.

**Graphical Abstract:**

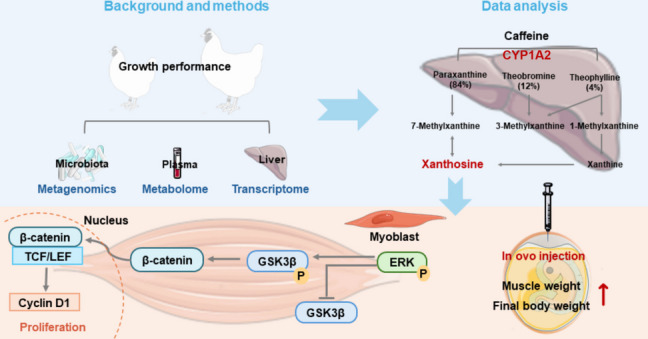

**Supplementary Information:**

The online version contains supplementary material available at 10.1186/s40104-025-01346-y.

## Background

Despite decades of intensive efforts, substantial variability in growth performance remains a persistent challenge in commercial broiler production. Even under standardized husbandry conditions and similar genetic backgrounds, marked disparities in growth efficiency persist, compromising production output [1], imposing significant economic losses, and increasing management complexity [2]. Growth performance is orchestrated by the intricate interplay of genetic potential, nutrient utilization, and environmental inputs, with metabolism serving as a central regulatory nexus. However, the molecular determinants underpinning divergent growth trajectories in broilers remain insufficiently defined. To address this knowledge gap, we employed an integrative multi-omics strategy to systematically elucidate the metabolic disparities underlying differential growth performance.

Skeletal muscle, the largest tissue mass in broilers and the most economically valuable product, is tightly regulated by systemic metabolic homeostasis. As the principal metabolic hub, the liver plays a pivotal role in coordinating nutrient fluxes and thereby modulating skeletal muscle development [3–5]. Hepatic amino acid synthesis and catabolism not only provide indispensable substrates for muscle protein accretion but also activate signaling cascades such as the mammalian target of rapamycin complex 1, which orchestrates myoblast proliferation, differentiation, and hypertrophy [6–9]. Although the contribution of hepatic metabolism to growth regulation has been increasingly investigated, its direct influence on skeletal muscle development remains incompletely understood.

Skeletal muscle growth proceeds through two distinct phases. During embryogenesis, myoblast proliferation establishes the total number of muscle fibers, whereas post-hatch development is primarily driven by fiber hypertrophy. In modern broilers characterized by accelerated growth, the embryonic stage has emerged as a particularly critical window for determining ultimate muscle mass. Previous studies employing in ovo administration of metabolites such as insulin-like growth factor-1, amino acids, or nutrients have demonstrated enhanced satellite cell proliferation and protein accretion, thereby improving growth performance post-hatch [10–12]. These findings provide compelling evidence that targeted metabolite supplementation during embryogenesis can effectively reprogram muscle development trajectories.

In the present study, growth performance was evaluated on the basis of body weight, and broilers representing high- and low-body-weight groups were selected from a large-scale commercial flock. Transcriptomic profiling of the liver was performed to capture functional shifts, while plasma metabolomics was leveraged to delineate systemic metabolic alterations. Here, we found that hepatic caffeine metabolism is markedly activated in high-growth broilers, leading to elevated circulating xanthosine. Although xanthosine has previously been implicated in the proliferation of adult stem cells, its role in skeletal myogenesis has remained largely unexplored [13–15]. To further investigate its potential regulatory function, we employed both in ovo and in vitro experimental models to examine the developmental and cellular effects of xanthosine. Functional validation revealed that xanthosine promotes myoblast proliferation by activating the extracellular signal-regulated kinase (ERK)/glycogen synthase kinase 3β (GSK3β)/β-catenin signaling cascade, thereby accelerating embryonic myogenesis and enhancing post-hatch muscle growth. These findings define a previously unrecognized liver–muscle metabolic axis and establish xanthosine as a key molecular effector linking hepatic metabolism to skeletal muscle development.

## Methods

### Experimental design and animal treatments

All animal procedures were performed in accordance with the Guide for the Care and Use of Laboratory Animals of the National Institutes of Health and were approved by the Northwest A&F University Committee for Laboratory Animal Management and Ethics Review (protocol number: 2023-DK-015; approval date: Jun 2024). All experimental animals used in this study were male. The 42-day-old Arbor Acres (AA) white-feathered broilers, were obtained from the commercial facility of Shaanxi Haobang Food Co., Ltd. (Xianyang, China). Based on body weight, the broilers were divided into two groups: a high body weight group (HBW) and a low body weight group (LBW). The broilers were reared at the Animal Husbandry Experimental Base of Northwest A&F University. Samples from HBW and LBW broilers were collected for cecal metagenomic, plasma metabolomic, and liver transcriptomic analyses. Fecal samples were also obtained for subsequent fecal microbiota transplantation (FMT) experiments. One-day-old AA chicks provided by Xi'an Dacheng Poultry Co., Ltd. (Xianyang, China) were randomly assigned to either the high-body-weight fecal microbiota transplantation group (High-FMT, *n* = 30) or the low-body-weight fecal microbiota transplantation group (Low-FMT, *n* = 30). The fecal samples were homogenized in phosphate-buffered saline (PBS; 1:6, w/v) and left to stand at 4 °C. The supernatant was filtered through sterile gauze to obtain the microbial suspension, which was then supplemented with 30% glycerol. The resulting mixture was aliquoted and stored at −80 °C. Each chicken from High-FMT and Low-FMT group received 1 mL of the corresponding fecal suspension by oral gavage daily for 28 consecutive days.

AA white-feathered broiler eggs used for injecting xanthosine into embryos were provided by Xi'an Dacheng Poultry Co., Ltd. (Xianyang, China), and eggs were selected for uniformity in size and weight (68–72 g). In ovo injections were performed at two developmental stages—the sixth and twelfth days of embryonic development. Xanthosine was dissolved in PBS (Servicebio, Wuhan, China), and three different doses were injected per egg: 0 (PBS-only group), 200 μg (low-dose group), and 500 μg (high-dose group). The experimental groups were labeled as E6-P, E6-L, E6-H, E12-P, E12-L, and E12-H. The control group (Con) was left untreated and consisted of 25 eggs, while each treated group contained 30 eggs. The eggs were incubated under standard conditions using an automatic incubator (Jiayu, Shandong, China) according to the automatic incubation program. After hatching, the chicks were transferred to the Animal Experiment Center at Northwest A&F University. The day when all the experimental chicks hatched was designated as d 1. In this experiment, the hatching rate of the chicks and the survival rate of the broilers during the 42-day rearing period are shown in Table S1.

Rearing conditions were maintained in strict accordance with the AA Broiler Management Handbook (Aviagen, Huntsville, UK). During the rearing period, broilers were provided ad libitum access to a corn-soybean meal-based diet and fresh water. Detailed feed formulations are provided in Table S2.

### Sample collection

HBW and LBW broilers used for omics sequencing were weighed at 42 days of age, and samples were collected at 43 days of age (HBW/LBW, *n* = 7). Broilers injected with xanthosine for embryo eggs were sampled at 28 and 42 days of age respectively, with 10 birds selected from each group at each time point (*n* = 10). Whole blood was added to tubes containing ethylenediaminetetraacetic acid anticoagulant, mixed upside down, fully anticoagulated, placed in a 37 °C water bath, centrifuged at 1,269 × *g* at room temperature for 15 min within 1 h, and the upper light yellow transparent liquid was taken as plasma, which was stored at −80 °C for testing. Tissue samples included breast muscle, leg muscle, liver, jejunum, ileum, cecum, and cecal contents. The liver, right breast muscle and right leg muscle were dissected for weighing, and the same position tissues on the left were frozen in liquid nitrogen and then stored at −80 °C for testing.

### Histological analysis

Liver, breast muscle, and leg muscle tissues were collected from consistent locations and fixed in 4% paraformaldehyde. Samples were subsequently embedded in paraffin, sectioned, dewaxed, and stained with hematoxylin and eosin (H&E, Servicebio, Wuhan, China) for histological analysis. Histological images were captured using an optical microscope (Nikon Eclipse Ni, Tokyo, Japan). The imaging results were statistically analyzed using Image J software.

### Metagenomic sequencing of cecal contents

Genomic DNA was extracted from samples using the Mag-Bind^®^ Soil DNA Kit (Omega Bio-tek, Norcross, Georgia, USA) following the manufacturer’s protocol. DNA concentration and purity were assessed with TBS-380 and NanoDrop 2000, respectively, and quality was verified via 1% agarose gel electrophoresis. DNA was fragmented to approximately 350 bp using the Covaris M220 for paired-end library preparation. Libraries were constructed with NEXTFLEX Rapid DNA-Seq (Bioo Scientific, Austin, Texas, USA) and sequenced on an Illumina NovaSeq 6000 platform (Illumina Inc., San Diego, California, USA) at Majorbio Bio-Pharm Technology Co., Ltd. (Shanghai, China). Data were analyzed on the Majorbio Cloud Platform; adaptor trimming and quality filtering were performed using fastp (length < 50 bp or quality < 20). High-quality reads were assembled with MEGAHIT v1.12, and contigs > 300 bp were selected for analysis. Gene abundance was quantified by aligning reads to a non-redundant gene set (95% identity) using SOAPaligner v2.21. Sequence data were deposited in the NCBI Short Read Archive (Accession No. PRJNA1331926).

### Non-targeted metabolome of plasma

Plasma samples (100 μL) were extracted with 400 μL acetonitrile:methanol (1:1, v/v) containing 0.02 mg/mL L-2-chlorophenylalanine (internal standard). After ultrasonic extraction (30 min at 5 °C and 40 kHz) and incubation at −20 °C for 30 min, the supernatant was collected by centrifuged (13,000 × g, 15 min, 4 °C), dried under a stream of nitrogen, and reconstituted in acetonitrile:water (1:1, v/v) for LC–MS/MS analysis. QC samples were prepared by pooling equal volumes of all samples and injected intermittently. Analysis was performed using a UHPLC-Q Exactive HF-X system (Thermo Fisher Scientific, Waltham, MA, USA) with separation on an HSS T3 column. Mass spectra were acquired in positive/negative modes (*m*/*z* 70–1,050, DDA mode). Data processing was performed using Progenesis QI, with spectral matching to HMDB, Majorbio, and an in-house database. Multivariate analysis was conducted in R, with model stability assessed by seven-fold cross-validation. Significant metabolites were identified based on VIP > 1.0 and *P* < 0.05, and pathway analysis was conducted using KEGG and Fisher’s exact test in Python. The metabolomics data have been deposited to MetaboLights repository with the study identifier MTBLS13036.

### Liver transcriptome analysis

Total RNA was extracted from liver tissue using TRIzol^®^ Reagent (Qiagen, Hilden, Germany) and assessed with a 5300 Bioanalyzer (Agilent Technologies, Santa Clara, CA, USA) and NanoDrop 2000. Samples with concentration ≥ 20 ng/μL, total RNA > 1 μg, and RQN > 4.5 were used for library construction. RNA sequencing libraries were prepared with the Illumina^®^ Stranded mRNA Prep, Ligation kit (1 μg input), size-selected (300–400 bp), and PCR-amplified (10–15 cycles), followed by quantification (Qubit 4.0) and sequencing on an Illumina NovaSeq 6000 (PE150). Raw reads were processed with fastp, aligned to the reference genome using HISAT2, and assembled with StringTie. Differential expression was analyzed with DESeq2 (|log_2_FC| ≥ 1, FDR < 0.05). KEGG enrichment of differentially expressed genes (DEGs) was performed with Fisher’s exact test (BH correction, FDR < 0.05) using the SciPy package in Python. All analyses were conducted on the Majorbio Cloud Platform. Sequence data were deposited in the NCBI Short Read Archive (Accession No. PRJNA1331257).

### Cell culture and stimulation

The murine myoblast cell line C2C12 was obtained from Pricella (Procell, Wuhan, China) and cultured in DMEM/F12 (Gibco, Grand Island, NY, USA) medium supplemented with 10% fetal bovine serum (Gibco, Grand Island, NY, USA). Cells were maintained under standard culture conditions at 37 °C in a humidified atmosphere containing 5% CO_2_. Xanthosine (Merck Millipore, Darmstadt, Germany) and SCH772984 (TargetMol, Boston, MA, USA) used in the cell experiments was dissolved in dimethyl sulfoxide (Solarbio, Beijing, China). SCH772984 was added at a final concentration of 1 μmol/L simultaneously with xanthosine, and cells were incubated for 24 h under standard culture conditions.

### Cell Counting Kit-8 (CCK-8) assay

For the CCK-8 assay, C2C12 cells were seeded into 96-well plates (10 replicate wells per group) and treated with 200 µL of xanthosine at concentrations of 0, 0.5, 1, 5, 10, or 50 μmol/L. Cells were incubated under standard culture conditions for 24 or 48 h. After incubation, the medium was replaced with 200 µL of serum-free medium containing CCK-8 reagent (10:1 dilution; Beyotime Biotechnology, Shanghai, China) and incubated at 37 °C for 1 h. Absorbance was then measured at 450 nm using a microplate reader.

### 5-Ethynyl-2′-deoxyuridine (EdU) assay

The EdU cell proliferation assay was performed using a commercial kit (Beyotime Biotechnology, Shanghai, China). C2C12 cells were seeded into 48-well plates (three technical replicates per group). After 24 h of stimulation, cells were incubated with EdU working solution (diluted according to the manufacturer’s instructions) at 37 °C for 2 h. Cells were then fixed with 4% paraformaldehyde and permeabilized with 100 µL of 0.3% Triton X-100 in PBS per well at room temperature. After washing three times with PBS, the staining solution was prepared as instructed and applied at room temperature in the dark. Hoechst 33342 solution (diluted in deionized water) was subsequently added and incubated under the same conditions. After final washes, cells were imaged with a fluorescence microscope (Nikon Eclipse Ni, Tokyo, Japan), and the percentage of EdU-positive cells was quantified using ImageJ software.

### Western blot analysis

After processing, the cells were placed on ice, washed with PBS, and lysed using 1 × loading buffer (Beyotime Biotechnology, Shanghai, China) diluted with RIPA lysis buffer. The samples were boiled and loaded. The proteins were separated by sodium dodecyl sulfate—polyacrylamide gel electrophoresis until the desired protein bands appeared and were transferred to a polyvinylidene fluoride membrane (Roche, Basel, Switzerland). Then, a 5% skim milk block was applied for 1 h at room temperature, followed by incubation of the primary antibody at 4 °C overnight and the secondary antibody at room temperature for 2 h. After each stage, the membrane was washed with TBST. Finally, ECL Plus (Thermo Fisher Scientific, Waltham, MA, USA) was used for imaging. The imaging results were statistically analyzed using Image J software. The following primary antibodies were used for detection: Cyclin D1 (mouse, Proteintech, China), PCNA (rabbit, Proteintech, China), p-AKT (Ser473, mouse, Proteintech, China), AKT (rabbit, Proteintech, China), p-mTOR (Ser2448, mouse, Proteintech, China), mTOR (mouse, Proteintech, China), p-Erk1/2 (Thr202/Tyr204, rabbit, CST, USA), Erk1/2 (rabbit, CST, USA), p-GsK3β (Ser9, rabbit, Selleck, USA), GSK3β (rabbit, Proteintech, China), β-Catenin (rabbit, Selleck, USA), GAPDH (rabbit, Proteintech, China).

### Statistical analysis

The sequencing analysis was conducted using the Illumina NovaSeq platform provided by Majorbio Bio-Pharm Technology Co., Ltd. (Shanghai, China). All data generated in this study are presented as mean ± standard error of the mean (SEM). Statistical analyses were performed using GraphPad Prism 8 (GraphPad Software, Inc., CA, USA) and SPSS software (IBM, Armonk, NY, USA). Depending on the experimental design, the Wilcoxon test, one-way analysis of variance (ANOVA), or Student’s *t*-test was used. Statistical significance compared to the control group is indicated as ^*^P < 0.05, ^**^P < 0.01 and ^***^P < 0.001. Lowercase letters (a, b, c) indicate *P* < 0.05, while uppercase letters (A, B, C) indicate *P* < 0.01. Values marked "ns" are not statistically significant.

## Results

### Divergent muscle phenotypes in high- and low-body-weight broilers

**Fig. 1:**
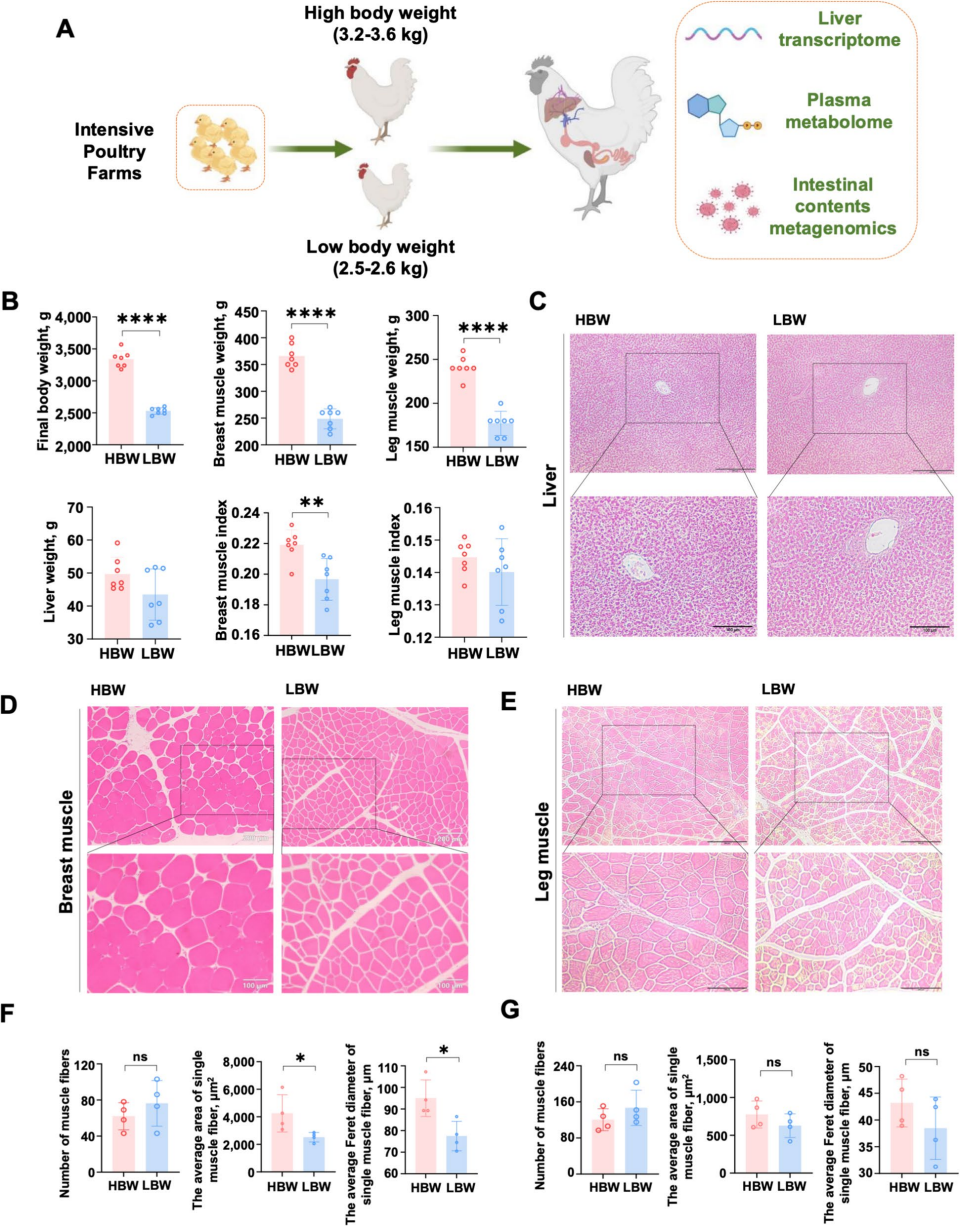
Phenotypic differences between high- and low-body-weight broilers. **A** Overview of multi-omics analysis comparing high-body-weight (HBW, 3.2–3.6 kg) and low-body-weight (LBW, 2.5–2.6 kg) broilers collected from intensive poultry farms. Cecal contents, plasma, and liver samples were subjected to metagenomic, non-targeted metabolomic, and transcriptomic profiling (*n* = 7). **B** Phenotypic data of high- and low-body-weight broilers, including final body weight, breast muscle weight, leg muscle weight, liver weight, breast muscle index and leg muscle index. **C–E** Representative images of HE-stained liver, breast muscle, and leg muscle tissues (upper panels, scale bar = 200 µm; lower panels, scale bar = 100 µm). **F** and** G** Histological analysis of breast and leg muscle fibers (per field at 100 × magnification), including fiber number, average area and average Feret diameter of single muscle fiber (*n* = 4). Data are presented as the mean ± SEM. Statistical analyses were performed using Student's *t*-tests (^*^P < 0.05, ^**^P < 0.01, ^***^P < 0.001)

To investigate the biological basis of growth disparities among broilers with comparable genetic backgrounds and standardized rearing conditions, AA white-feathered broilers were selected at market age from a large-scale commercial flock (Fig. 1A). Broilers were stratified into HBW and LBW groups. Body weights differed markedly between HBW (3,180–3,570 g, *n* = 7) and LBW (2,450–2,600 g, *n* = 7) groups, validating the stratification criteria (Fig. 1B). Phenotypic data revealed that HBW broilers exhibited significantly greater breast muscle mass and an elevated breast muscle index (breast mass-to-body-weight ratio) relative to LBW counterparts (Fig. 1B). Although leg muscle mass was higher in HBW broilers, the leg muscle index did not differ appreciably between groups (Fig. 1B). Liver parameters, including organ weight and histological architecture, showed negligible differences; H&E staining confirmed the absence of pathological lesions or abnormal lipid accumulation in both groups (Fig. 1C).

Histological and morphometric analyses of breast and leg muscles were conducted to characterize the observed phenotypic divergence (Fig. 1D, E). Total muscle fiber number did not differ between groups (Fig. 1F, G). However, breast muscle fibers in HBW broilers exhibited markedly increased diameters and cross-sectional areas, whereas leg muscle fiber morphology remained comparable (Fig. 1F, G).

### Minimal cecal microbiota divergence with negligible impact on growth performance

**Fig. 2:**
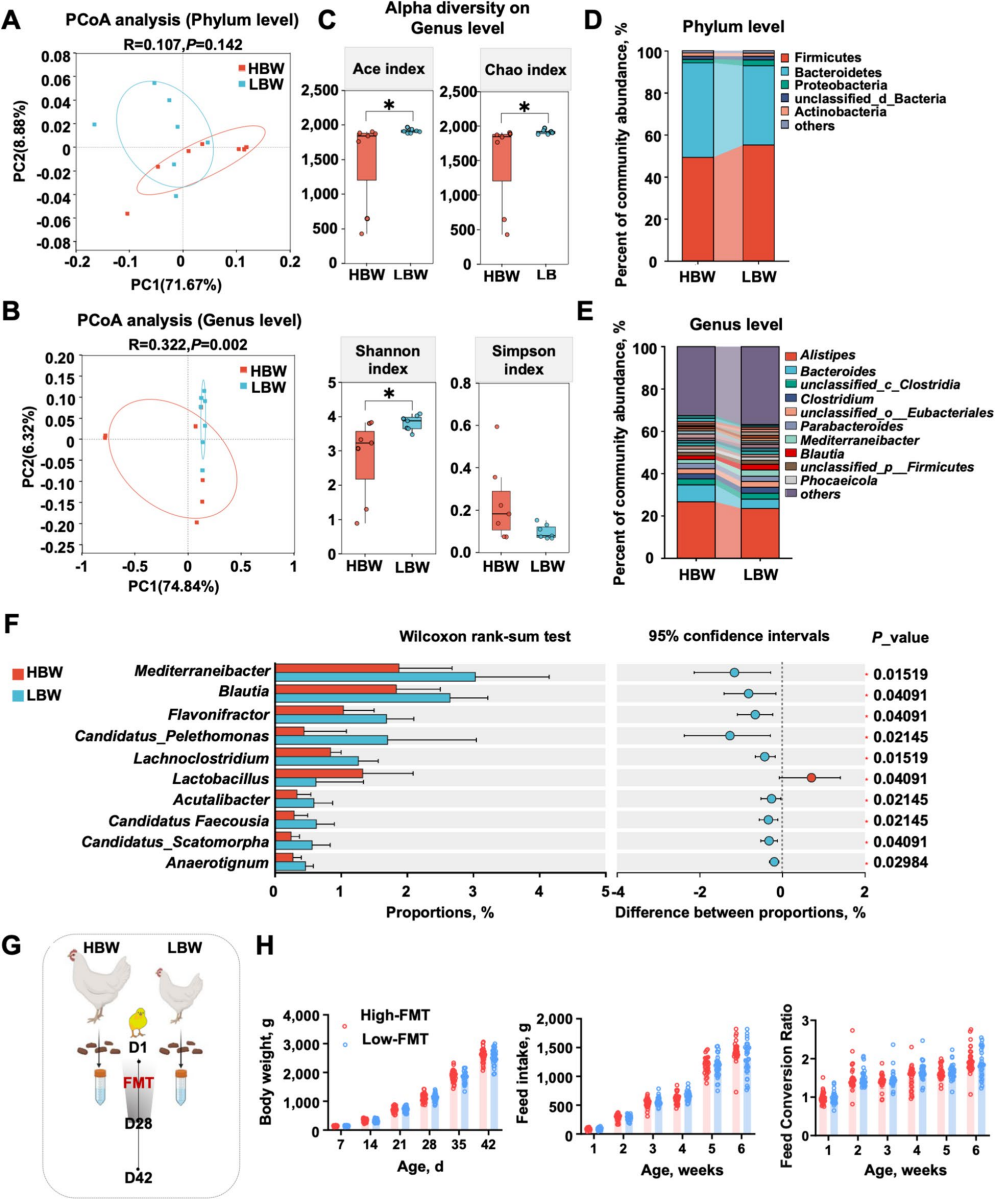
Subtle differences in the cecal microbiota between high- and low-body-weight broilers. **A** and **B** PCoA analysis at the phylum level and genus level. **C** Alpha diversity indices (Ace index, Chao index, Shannon index and Simpson index) at the genus level. **D** and** E** Community composition bar plots at the phylum and genus levels. **F** The top 10 differentiating bacterial genera at the genus level. Statistical significance was determined using the Wilcoxon rank sum test (^*^P < 0.05, ^**^P < 0.01, ^***^P < 0.001).** G** Schematic diagram of the experimental design of FMT: fresh feces from broilers with high- and low- body weight broilers were homogenized into suspensions and orally administered to 1-day-old chicks by daily gavage for 28 consecutive days, followed by standard rearing until 42 d. The receptor chickens were divided into two groups, namely High-FMT and Low-FMT (*n* = 30). **H** Effects of FMT on production performance, including body weight, feed intake, and feed conversion ratio. Data are presented as the mean ± SEM. Statistical analyses were performed using Student's *t*-tests

The gut microbiota is a recognized modulator of growth in poultry [16–18]. To assess whether intestinal microbial composition contributes to body weight variation, metagenomic sequencing of cecal contents from HBW and LBW broilers was performed. Principal coordinates analysis (PCoA) at the phylum level showed negligible variation in β-diversity (Fig. 2A). At the genus level, β- and α-diversity metrics, including Ace, Chao, and Shannon index, exhibited modest but statistically detectable differences (Fig. 2B, C). Taxonomic profiling indicated that Firmicutes and Bacteroidetes dominated the cecal community, together comprising over 90% of total abundance, with minimal intergroup divergence (Fig. 2D). *Alistipes* and *Bacteroides* were the predominant genera (~ 20% and ~ 6%, respectively), while all other genera individually represented < 5% of the community (Fig. 2E). Importantly, neither *Alistipes* nor *Bacteroides* displayed appreciable differences between HBW and LBW broilers. Linear discriminant analysis identified the top 10 genera with intergroup differences, among which *Lactobacillus* was significantly enriched in the HBW group (Fig. 2F). Functional profiling of the cecal microbiota showed that pathways related to glycan biosynthesis and metabolism, biosynthesis of secondary metabolites, and metabolism of terpenoids and polyketides were more represented in HBW birds (Fig. S1A). The correlation heatmap displays the associations between the relative abundance of major cecal microbial taxa and the concentrations of identified metabolites (Fig. S1B).

To determine whether these subtle compositional shifts could causally influence growth, FMT was performed. Cecal contents from HBW or LBW donors were orally administered to one-day-old AA broilers, which were reared for 42 d (Fig. 2G). Throughout the experimental period, body weight, feed intake, and feed conversion ratio were comparable between recipient groups (Fig. 2H), suggesting that overall microbial composition exerted limited effects on growth performance under these conditions.

### Amino acid and caffeine metabolism underlie plasma metabolic divergence between HBW and LBW broilers

**Fig. 3:**
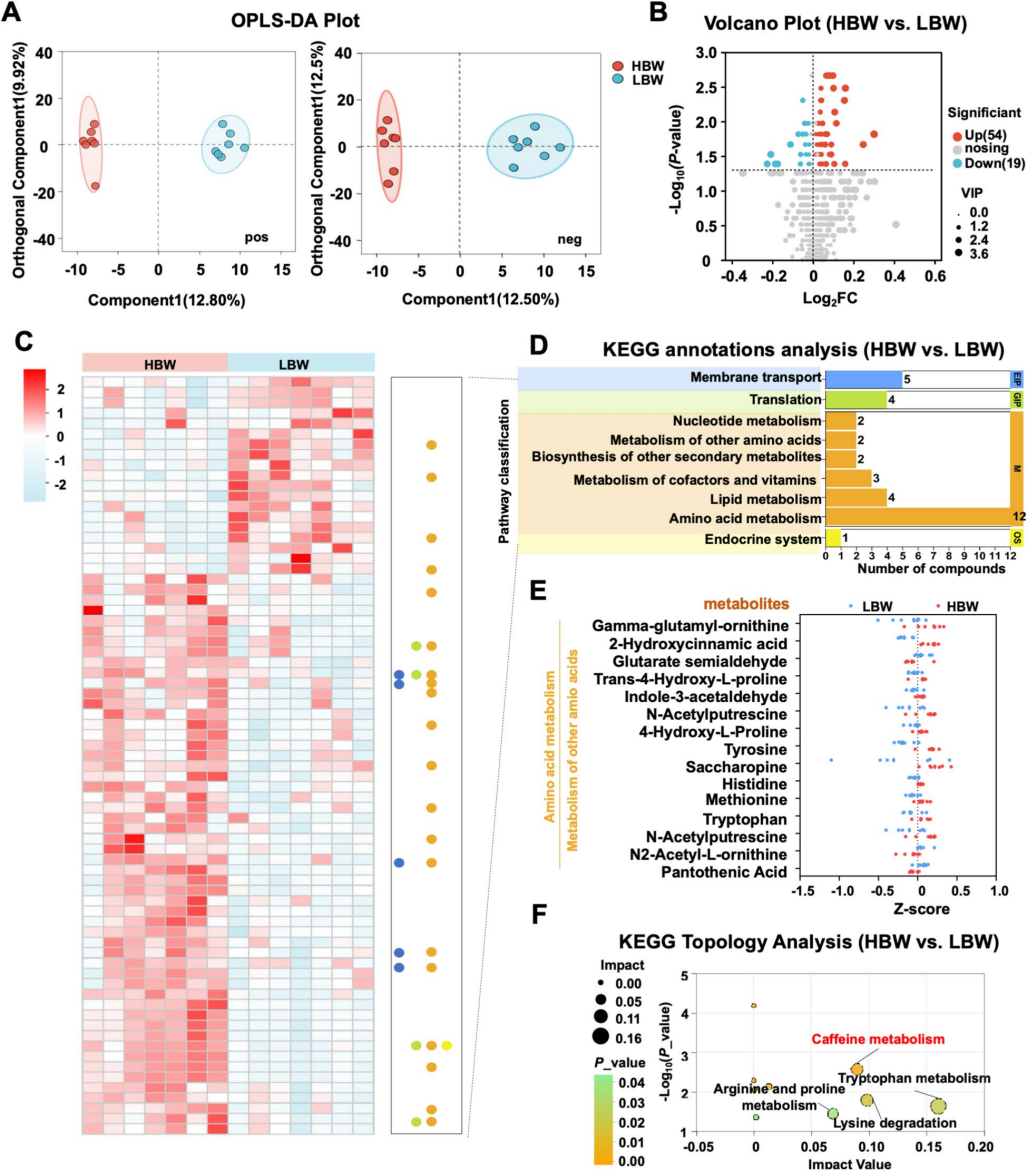
Divergence in body weight is associated with altered amino acid metabolism and caffeine metabolism.** A** OPLS-DA plots of plasma metabolites from HBW and LBW groups. Positive ions are shown on the left, while negative ions are shown on the right.** B** Volcano plot showing differential metabolites in plasma between HBW and LBW. **C** Heatmap of significantly difference plasma metabolites. **D** KEGG annotation analysis displaying secondary pathway categories enriched by the SCMs. The *x*-axis represents the number of metabolites mapped to each pathway, while different colors indicate distinct classes of metabolic pathways.** E** Scatter plot of differential metabolites enriched in amino acid metabolism and related sub-pathways. **F** KEGG topological pathway analysis, where the *x*-axis indicates pathway impact (reflecting the relative importance of each metabolite within the pathway), and the *y*-axis represents pathway enrichment significance expressed as −log_10_ (*P*_value)

Broiler metabolic capacity constitutes a key determinant of growth performance. To gain an in-depth understanding of systemic metabolic status, we next investigated systemic metabolic differences via untargeted plasma metabolomics. Orthogonal partial least-squares discriminant analysis (OPLS-DA) demonstrated a clear separation of metabolic profiles between the two groups (Fig. 3A). Among the detected metabolites, 73 were classified as significantly changed metabolites (SCMs), of which 54 were upregulated and 19 downregulated in HBW broilers relative to LBW counterparts (Fig. 3B).

Pathway enrichment analysis revealed that SCMs were predominantly associated with amino acid metabolism, which emerged as the most substantially altered category (Fig. 3C, D). Collectively, amino acid metabolism and related pathways encompassed 15 differentially abundant metabolites. In HBW broilers, levels of key amino acids, including tyrosine, histidine, tryptophan, and methionine, were elevated, accompanied by amino acid-derived metabolites such as indole-3-acetaldehyde, γ-glutamyl-ornithine, and 2-hydroxycinnamic acid (Fig. 3E).

Notably, KEGG topology analysis identified caffeine metabolism as the most significantly perturbed subpathway, highlighting its potential central role in mediating growth-related metabolic differences (Fig. 3F). These results indicate that enhanced amino acid and caffeine metabolism collectively contribute to the systemic metabolic phenotype associated with high-body-weight broilers.

### Hepatic caffeine metabolism identified as a central growth-regulatory pathway

**Fig. 4:**
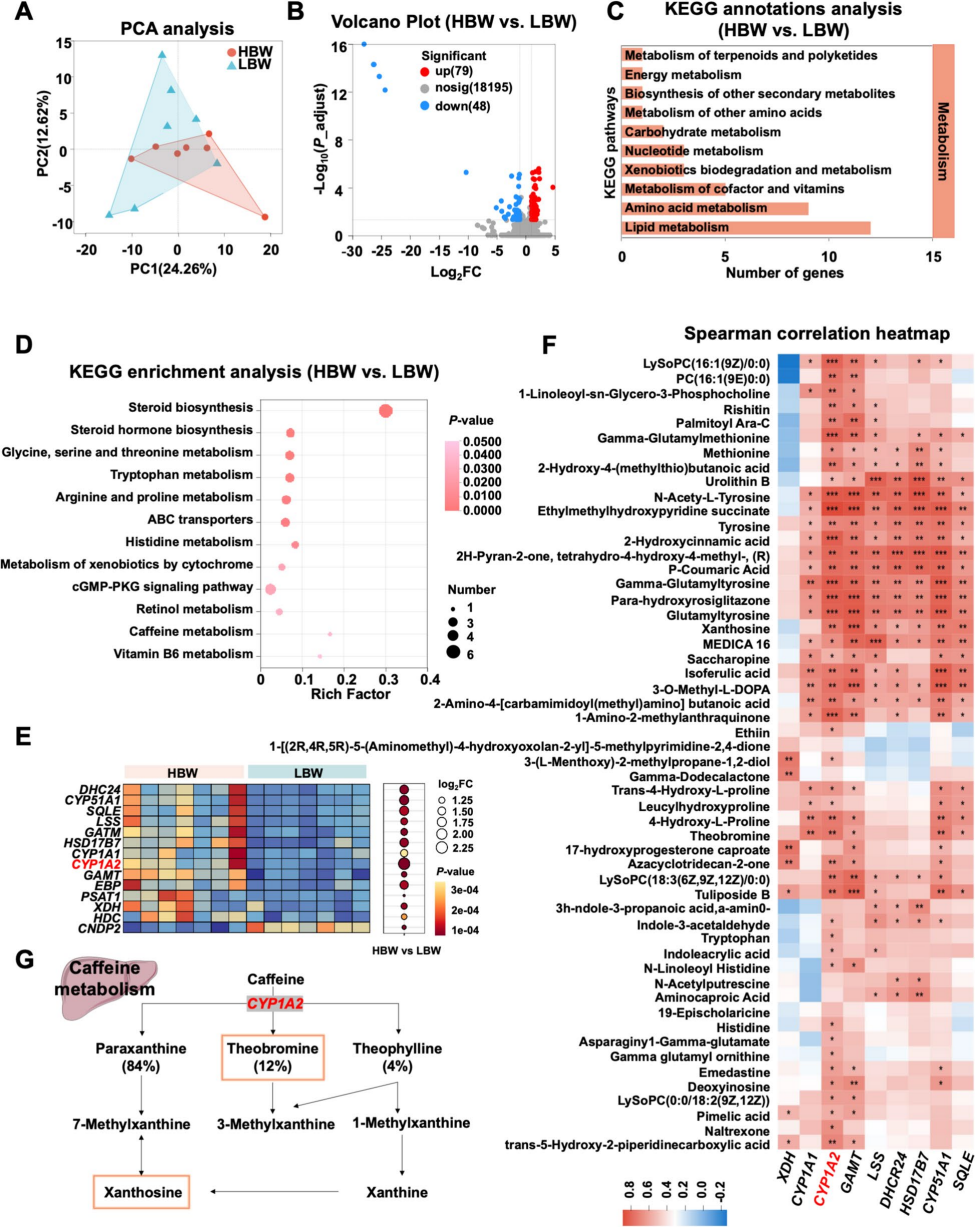
Caffeine metabolism is significantly enriched in both the plasma metabolome and hepatic transcriptome. **A** PCA was performed on liver samples from the HBW and LBW broilers. **B** Volcano plot of DEGs in liver samples between HBW and LBW broilers. **C** KEGG annotation analysis displaying secondary pathway categories enriched by the DEGs. **D** KEGG enrichment analysis. The horizontal axis represents the Rich factor. The larger the Rich factor, the greater the degree of enrichment. The size of the points indicates the number of genes in this pathway. **E** Heatmap of the main DEGs related to amino acid metabolism and lipid metabolism. The size of the circle represents the log_2_FC, and the darker color represent the smaller *P*-value. **F** Spearman correlation heatmap between SCMs in plasm and the main DEGs in liver. Each cell in the heatmap represents the correlation between a metabolite and an associated trait, with different colors indicating the magnitude of the correlation coefficient. Correlations were calculated using Spearman’s rank correlation. The asterisk indicates the magnitude of the significant *P-*value (^*^P < 0.05, ^**^P < 0.01, ^***^P < 0.001). **G** Metabolic pattern of caffeine in the liver. The metabolites within the red box showed a significant increase in the plasma metabolome, and the highlighted gene was significantly upregulated in the liver transcriptome

The pronounced enhancement of amino acid metabolism in HBW broilers underscores systemic metabolic reprogramming between groups. Given the liver’s pivotal role in maintaining metabolic homeostasis, it likely represents a key determinant of overall growth performance. To delineate hepatic contributions, RNA sequencing was performed on liver tissue from HBW and LBW broilers. Principal component analysis (PCA) revealed clear segregation of hepatic transcriptomic profiles between the two groups (Fig. 4A).

A total of 127 DEGs were identified, including 79 upregulated and 48 downregulated in HBW relative to LBW broilers (Fig. 4B). KEGG functional annotation indicated that DEGs were predominantly enriched in pathways associated with lipid and amino acid metabolism (Fig. 4C). Notably, caffeine metabolism emerged as a significantly enriched tertiary pathway, consistent with plasma metabolomic findings (Fig. 4D).

Among DEGs related to amino acid and lipid metabolism, cytochrome P450 family 1 subfamily A member 2 (*CYP1A2*), encoding the rate-limiting enzyme in caffeine catabolism, was markedly upregulated in HBW broilers, exhibiting a fold-change substantially exceeding that of other DEGs (Fig. 4E). Correlation analysis between key hepatic DEGs and plasma SCMs revealed a strong positive association between *CYP1A2* expression and circulating levels of xanthosine and theobromine (Fig. 4F), indicating a potential mechanistic link between hepatic caffeine metabolism and enhanced growth performance (Fig. 4G).

### In ovo xanthosine administration enhances muscle mass and growth performance in broilers

Integrative analyses of plasma metabolomics and hepatic transcriptomics identified the caffeine metabolic pathway as a key determinant of body weight-associated phenotypic divergence. The terminal product of this pathway, xanthosine, was markedly elevated in the plasma of HBW broilers, suggesting a potential role in promoting growth and skeletal muscle development. Although xanthosine has been reported to stimulate cellular proliferation [13, 15], its specific function in avian skeletal muscle remained unexplored. Given that myoblast proliferation predominantly occurs during embryogenesis, we administered xanthosine via in ovo injection at high or low doses into the air sac of fertilized Arbor Acres broiler eggs.

**Fig. 5:**
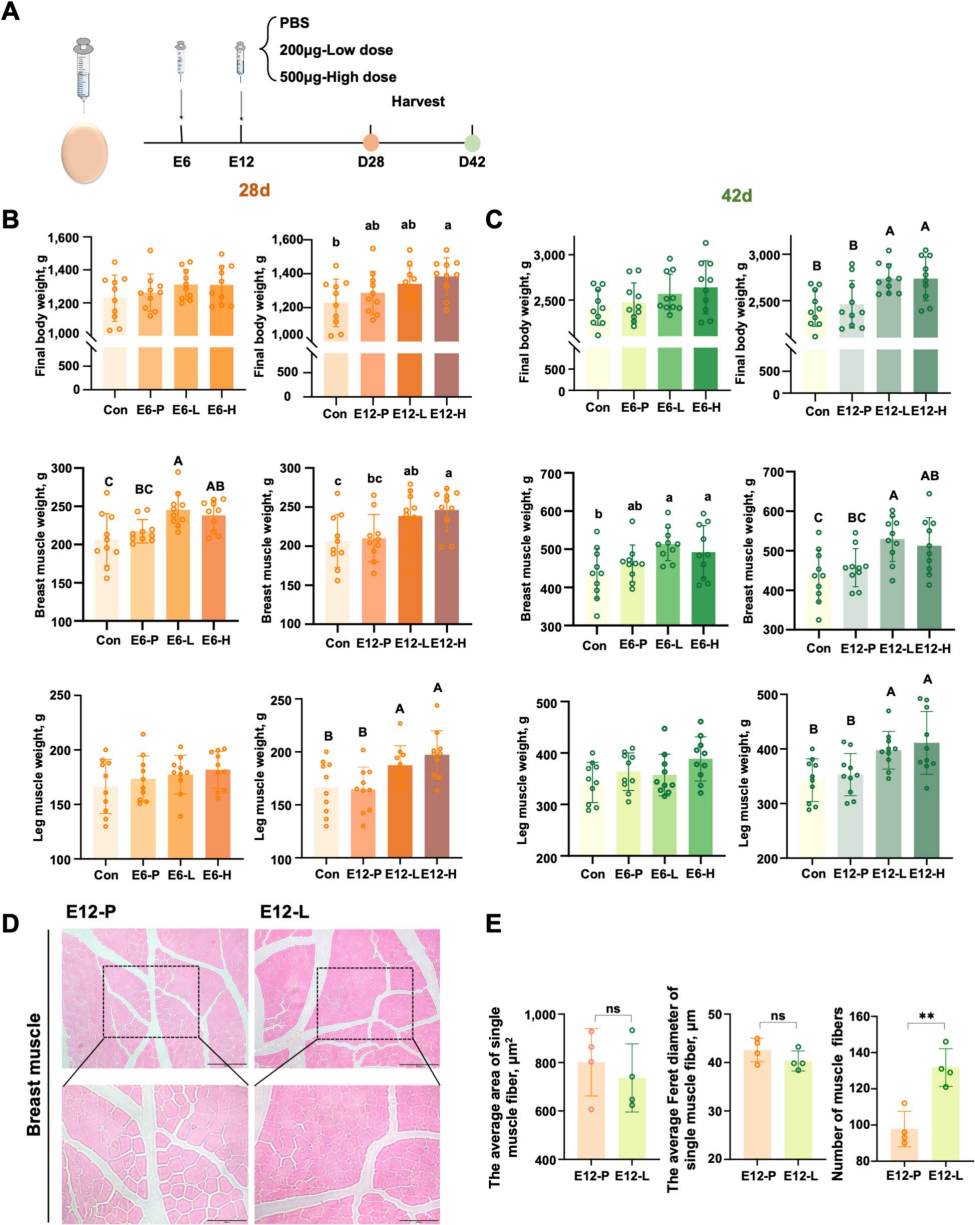
In ovo injection of xanthosine enhances growth performance in broilers.** A** Schematic representation of the experimental design for in ovo injection of xanthosine. A total of 205 AA white-feathered broiler eggs (68–72 g) were used. At E6 or E12, eggs were injected with either PBS (0 μg, E6-P/E12-P), a low dose of xanthosine (200 μg, E6-L/E12-L), or a high dose of xanthosine (500 μg, E6-H/E12-H). An untreated group served as the control (Con). Ten broiler chickens were sampled from each group at the two-day-old stage. **B** and **C** Effects of embryonic xanthosine injection on body weight, breast muscle mass, and leg muscle mass at 28 d and 42 d post-hatch. Data were analyzed by one-way ANOVA. Groups labeled with different lowercase letters (a, b, c) indicate *P* < 0.05, whereas those with different uppercase letters (A, B, C) indicate *P* < 0.01. **D** Representative images of HE-stained breast muscle tissues from the E12-P and E12-L groups (upper panels, scale bar = 200 µm; lower panels, scale bar = 100 µm; *n* = 4). **E** Histological analyses of breast muscle fibers at 100 × magnification included muscle fiber number, average cross-sectional area, and mean Feret diameter per field (*n* = 4). Data are presented as the mean ± SEM. Statistical analyses were performed using Student's *t*-tests (^*^P < 0.05, ^**^P < 0.01, ^***^P < 0.001)

Injections were performed at embryonic day (E) 6 or E12, with subsequent sampling at post-hatch 28 d and 42 d (Fig. 5A). At 28 d, E6 administration significantly increased breast muscle mass, while final body weight and leg muscle mass remained unchanged. In contrast, E12 injection led to significant increases in both final body weight and muscle mass, encompassing pectoral and leg muscles (Fig. 5B). These enhancements persisted through 42 d, mirroring the patterns observed at 28 d (Fig. 5C). Notably, no significant differences were observed between low- and high-dose treatments.

Histological analysis of breast muscle from E12-treated groups at 42 d revealed a pronounced increase in muscle fiber number in the E12-L group, without significant changes in fiber diameter or cross-sectional area compared with controls (Fig. 5D, E), indicating enhanced myogenesis. These findings suggest that xanthosine promotes skeletal muscle development primarily by increasing myoblast proliferation.

### Xanthosine promotes myoblast proliferation via ERK/GSK3β/β-catenin signaling

**Fig. 6:**
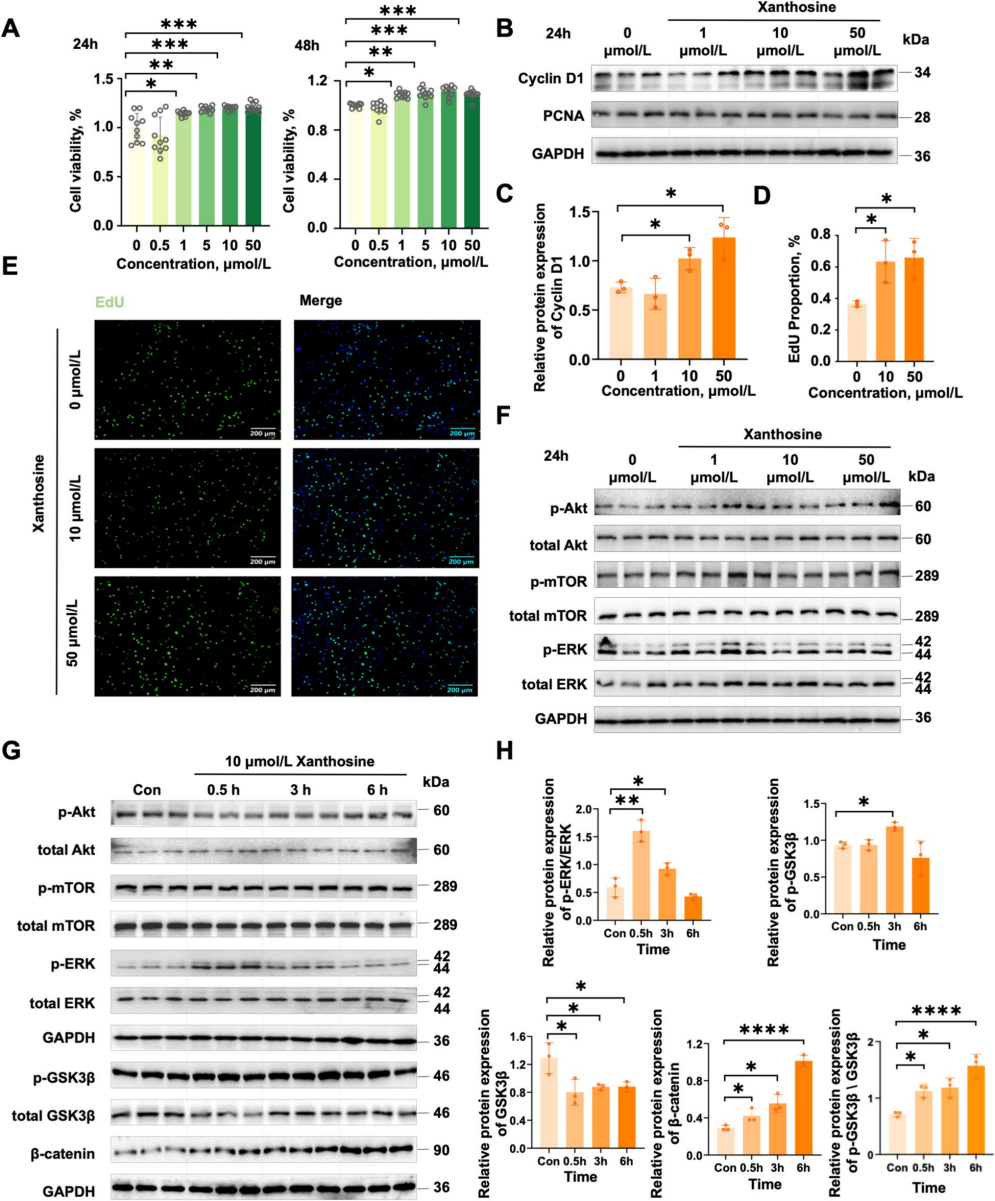
Xanthosine promotes the proliferation of myoblasts.** A** CCK-8 assay assessing the effects of different concentrations of xanthosine on myoblast proliferation at 24 h and 48 h. **B** Western blot analysis of Cyclin D1 and PCNA protein levels in C2C12 cells following xanthosine treatment after 24 h, with GAPDH used as the control. **C** Relative protein abundance of Cyclin D1 (*n* = 3). **D** and **E** Representative EdU fluorescence staining images of C2C12 cells (*n* = 3).** F** Western blot analysis of Akt-mTOR and ERK signaling pathways in C2C12 cells following xanthosine treatment after 24 h. **G** and **H** Western blot analysis of Akt-mTOR, ERK, Wnt/β-catenin signaling pathways (p-Akt, Akt, p-mTOR, mTOR, p-ERK, ERK, p-GSK3β, GSK3β, and β-catenin) in C2C12 cells under short-term xanthosine stimulation (*n* = 3). Data are presented as the mean ± SEM. Statistical analyses were performed using Student's *t*-tests (^*^P < 0.05, ^**^P < 0.01, ^***^P < 0.001)

We next sought to delineate the cellular and molecular mechanisms through which xanthosine exerts its pro-myogenic effects. Mechanistic studies were performed in C2C12 myoblasts. CCK-8 assays demonstrated that graded concentrations of xanthosine significantly increased myoblast proliferation at both 24 and 48 h (Fig. 6A). Based on these results, 10 μmol/L and 50 μmol/L were selected for subsequent analyses. After 24 h of treatment, Cyclin D1 expression was markedly upregulated (Fig. 6B, C), and the proportion of EdU-positive cells increased by ~ 30% (Fig. 6D, E), confirming the proliferative effect of xanthosine. To identify the signaling pathways mediating this response, we examined phosphorylation of canonical regulators of muscle growth, including Akt, mTOR, and ERK. No activation was detected at 24 h (Fig. 6F). Given the transient kinetics of ERK and Akt phosphorylation, earlier time points were assessed. At 30 min post-treatment, ERK phosphorylation was strongly induced and subsequently declined, whereas Akt and mTOR phosphorylation remained unchanged (Fig. 6G, H). Because the Wnt/β-catenin axis plays a pivotal role in myogenesis, we further evaluated its core components. At 30 min after xanthosine stimulation, total GSK3β protein levels were reduced, accompanied by increased GSK3β phosphorylation and robust β-catenin accumulation (Fig. 6G, H). These findings indicate that xanthosine activates ERK and converges on the GSK3β/β-catenin signaling cascade, thereby promoting myoblast proliferation.

**Fig. 7:**
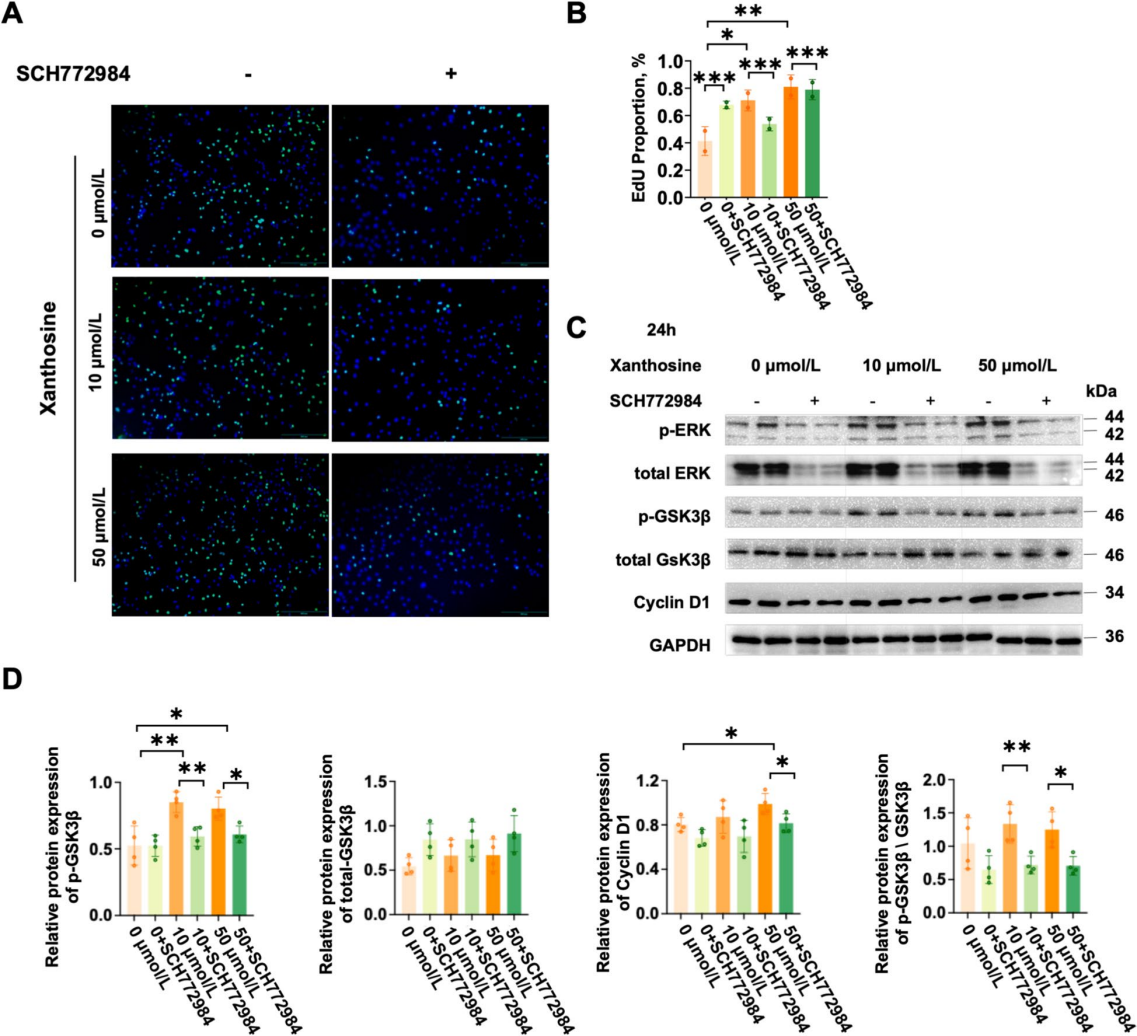
Xanthosine promotes myoblasts proliferation by activating the ERK/GSK3β/β-catenin pathway. **A** Representative EdU fluorescence staining images of C2C12 cells treated with or without the ERK inhibitor SCH772984 for 24 h. **B** Quantification of EdU-positive cells (*n* = 4). **C** and **D** Western blot analysis of ERK and Wnt/β-catenin signaling components, including p-ERK, ERK, p-GSK3β, GSK3β, and β-catenin, in C2C12 cells (*n* = 4). Data are presented as the mean ± SEM. Statistical analyses were performed using Student's *t*-tests (^*^P < 0.05, ^**^P < 0.01, ^***^P < 0.001)

To further investigate the relationship between ERK and Wnt signaling, ERK1/2 was pharmacologically inhibited using SCH772984. ERK inhibition abolished the xanthosine-induced proliferative effect (Fig. 7A, B), fully reversing the upregulation of Cyclin D1 and p-GSK3β, as well as the reduction in total GSK3β protein levels (Fig. 7C, D). These results demonstrate that ERK acts upstream of Wnt signaling to mediate xanthosine-driven myoblast proliferation. These findings establish an ERK/GSK3β/β-catenin signaling axis as the mechanistic conduit for xanthosine-induced myogenic proliferation.

## Discussion

Enhancing carcass yield remains a central objective in modern broiler production. Because growth performance is intrinsically constrained by systemic metabolic capacity, we employed a multi-omics framework to interrogate hepatic transcriptomes, plasma metabolomes, and cecal microbiota composition in HBW and LBW broilers. Microbial profiling revealed only subtle differences between groups, and FMT confirmed that these variations were insufficient to alter growth trajectories. By contrast, integrative analyses uncovered a pronounced enrichment of the caffeine metabolism pathway in HBW broilers. This pathway, predominantly hepatic and mediated by *CYP1A2*, was strongly upregulated at the transcriptional level. Xanthosine, a differentially abundant metabolite within this pathway, markedly promoted body weight gain and enhanced pectoral and leg muscle accretion when administered in ovo. Mechanistically, xanthosine activated the ERK/GSK3β/β-catenin signaling cascade, thereby driving myoblast proliferation. Collectively, these findings establish hepatic caffeine metabolism as a previously unrecognized determinant of skeletal muscle growth and highlight embryonic xanthosine supplementation as a feasible metabolic intervention to improve broiler production efficiency.

Recent studies have demonstrated that FMT administered during critical windows of intestinal immaturity can accelerate microbial maturation, enhance beneficial metabolite production, stabilize immune homeostasis, and ultimately improve growth performance [19–22]. Perturbations of the intestinal microbiota have likewise been shown to exert profound effects on host growth [17, 23–25]. To further investigate this relationship, we compared the cecal microbial communities of HBW and LBW broilers. Our analysis revealed negligible variation at the phylum level and several genera displayed measurable intergroup variation. FMT experiments revealed no significant differences between groups derived from HBW and LBW donors. This finding is consistent with a previous study, which showed that while FMT from HBW donors promoted growth relative to untreated controls, there were no detectable differences between HBW- and LBW-derived FMT groups [26]. We speculate that these minimal differences likely reflect the highly similar genetic background of AA broilers and their shared intensive rearing environment.

Notably, *Lactobacillus* exhibited an approximately twofold increase in HBW broilers. This trend aligns with previous observations showing that high-feed-efficiency chickens harbor elevated cecal *Lactobacillus* compared with low-efficiency broilers. Functional predictions further indicated that the cecal microbiota of HBW broilers displayed an enhanced metabolic capacity for carbohydrate utilization and plant-derived dietary substrates. The positive correlations between *Lactobacillus* abundance and multiple aromatic amino acid-derived and phenolic metabolites suggest an increased potential for the liberation and biotransformation of plant- and host-derived glycans and polyphenols. Similarly, the negative correlations with metabolites associated with inflammation and oxidative stress imply that elevated *Lactobacillus* levels may contribute to a more anti-inflammatory and metabolically favorable systemic milieu. The evidence indicates that direct oral administration of *Lactobacillus* at doses around 10^8^ CFU can confer measurable physiological benefits [27–29]. The absence of detectable *Lactobacillus*-associated effects in our FMT experiments may therefore reflect insufficient transfer of *Lactobacillus* at biologically effective levels through FMT. Nevertheless, the increased abundance of *Lactobacillus* and other putative probiotic taxa in HBW broilers underscores their potential functional relevance, warranting targeted mechanistic investigation in future studies.

The metabolic capacity of an organism is a critical determinant of growth performance [30]. To evaluate whether metabolic potential differed between HBW and LBW broilers, we performed untargeted plasma metabolomic profiling. HBW broilers exhibited a pronounced enhancement of amino acid metabolism, reflected by significantly elevated circulating amino acid levels. Beyond serving as substrates for myofibrillar protein synthesis, amino acids also function as signaling mediators that facilitate myogenesis, thereby supporting rapid muscle accretion [31]. Consistent with this metabolic signature, HBW broilers displayed markedly greater breast muscle mass and pectoral muscle index. Thus, the systemic upregulation of amino acid metabolism in HBW broilers likely represents a metabolic adaptation that underpins accelerated muscle proliferation and overall growth. At the same time, elevated xanthosine levels observed in HBW broilers. Xanthosine, as an intermediate in de novo purine biosynthesis, directly contributes to the synthesis of adenine and guanine nucleotides, thereby supporting DNA replication and cell proliferation [32, 33]. In addition, xanthosine can be metabolically linked to caffeine degradation through xanthine and other related intermediates, thereby participating in the regulation of caffeine metabolism. Although its abundance is relatively low, *Lactobacillus*, which is significantly enriched in the HBW group, has been shown in previous studies to modulate host purine and nucleotide metabolism [34, 35]. Moreover, caffeine and its derivatives have been reported to influence *Lactobacillus* abundance, suggesting that exposure to methylxanthines could further contribute to microbiota-associated metabolic differences [36]. Although previous studies reported a negative correlation between caffeine and *Lactobacillus* abundance [37], we suggest that this discrepancy may be related to enhanced *CYP1A2*-mediated caffeine degradation.

More importantly, caffeine metabolism emerged as the most significantly enriched pathway across both the hepatic transcriptome and plasma metabolome. This pathway is predominantly mediated by CYP1A2, which also contributes to tryptophan catabolism, lipid turnover, and oxidative stress regulation [38–41]. As the central site of caffeine catabolism, the liver metabolizes caffeine primarily via CYP1A2 [42], sequentially generating paraxanthine, theobromine, and theophylline, which are further degraded to xanthine and ultimately enter the purine degradation cascade to yield uric acid [43]. At the same time, CYP1A2 functions as an oxidase that catalyzes the demethylation of methylated molecules—particularly caffeine—releasing formaldehyde as a byproduct [44, 45]. The resulting formaldehyde is subsequently incorporated into the folate-dependent one-carbon metabolic pathway [46]. Enhanced one-carbon metabolism promotes nucleotide biosynthesis, lipid metabolism, and methionine regeneration [47], which is consistent with our metabolomic findings and may provide a mechanistic explanation for the accelerated muscle growth observed in broiler chickens. Notably, *CYP1A2* was the most strongly induced hepatic gene in HBW broilers, underscoring the role of hepatic caffeine metabolism as a key molecular determinant of growth performance.

In poultry, the total number of skeletal muscle fibers is fixed during embryogenesis [48]. Primary myofibers are established between E4 and E7, providing the structural scaffold of muscle, whereas secondary myofibers—generated between E8 and E16 through extensive progenitor proliferation—ultimately determine the final fiber number [48–50]. To evaluate the developmental role of xanthosine, we performed in ovo injections at E6 and E2, corresponding to the formation of primary and secondary myofibers, respectively. Consistent with this developmental trajectory, intervention during the secondary myofiber stage elicited more pronounced enhancements in muscle growth compared with the primary stage, highlighting the primacy of timing over dosage. Xanthosine administration significantly increased the number of pectoralis major fibers without affecting fiber diameter or cross-sectional area. Complementary in vitro assays further corroborated these findings, demonstrating that xanthosine promotes myoblast proliferation. Collectively, these results indicate that xanthosine primarily acts during embryogenesis to expand myofiber number, thereby establishing a larger myogenic foundation for post-hatch muscle growth.

In subsequent mechanistic analyses, xanthosine treatment markedly enhanced ERK phosphorylation, a canonical signaling cascade that drives myoblast proliferation [51, 52]. Concurrently, activation of the Wnt/β-catenin axis—an essential regulator of skeletal muscle development and regeneration—was detected, consistent with its established role in maintaining the proliferative capacity of myoblasts and progenitor cells [53–55]. Previous studies have demonstrated that Wnt overexpression significantly increases muscle mass in chicken embryos [56], raising the possibility that xanthosine promotes skeletal muscle growth through coordinated activation of the ERK/GSK3β/β-catenin signaling network, thereby stimulating robust expansion of myogenic precursor populations. Notably, pharmacological inhibition of ERK using the selective ERK1/2 inhibitor SCH772984 not only suppressed ERK phosphorylation but also reduced total ERK protein abundance. This unexpected reduction may reflect secondary regulatory mechanisms elicited by sustained pathway inhibition, such as alterations in protein stability or transcriptional feedback [57]; however, further investigation is required to delineate the precise underlying processes.

## Conclusions

This study identifies xanthosine, a hepatic caffeine metabolite, as a potent enhancer of skeletal muscle growth in broilers. Embryonic xanthosine administration significantly increased body weight and muscle mass at slaughter. Mechanistic analyses demonstrate that xanthosine promotes myoblast proliferation through activation of ERK/GSK3β/β-catenin signaling. These findings reveal a previously unappreciated liver–muscle metabolic axis and suggest xanthosine as a promising metabolic intervention to improve broiler production. Future work should investigate its impact on muscle-specific metabolic reprogramming, fiber type composition, and interactions with other anabolic pathways.

## Supplementary Information

Additional file 1: Table S1. Effects of in ovo xanthosine injection on hatching rate and post-hatch. Table S2. Composition of diets during the experiment.

Additional file 2: Fig. S1. Functional differences and cecal microbiota–metabolite associations between HBW and LBW broilers.

Additional file 3: Raw date of Western blot.

## Data Availability

The datasets produced or analyzed during the current study are available from the corresponding author on reasonable request.

## Abbreviations

AA: Arbor Acres
Akt: Protein kinase B
ANOVA: One-way analysis of variance
CCK-8: Cell Counting Kit-8
CYP1A2: Cytochrome P450 family 1 subfamily A member 2
DEGs: Differentially expressed genes
E: Embryonic day
EdU: 5-Ethynyl-2′-deoxyuridine
ERK: Extracellular regulated protein kinases
FMT: Fecal microbiota transplantation
GAPDH: Glyceraldehyde-3-phosphate dehydrogenase
GSK3β: Glycogen synthase kinase-3β
H&E: Hematoxylin–eosin
HBW: High body weight
High-FMT: High-body-weight fecal microbiota transplantation group
LBW: Low body weight
Low-FMT: Low-body-weight fecal microbiota transplantation group
mTOR: Mammalian target of rapamycin
OPLS-DA: Orthogonal partial least squares discriminant analysis
PBS: Phosphate-buffered saline
PCA: Principal component analysis
PCoA: Principal coordinates analysis
SCMs: Significantly changed metabolites
SEM: Standard error of the mean
Wnt: Wingless/Integrated
